# Evaluation of three commercially-available chikungunya virus immunoglobulin G immunoassays

**DOI:** 10.26633/RPSP.2017.62

**Published:** 2017-07-05

**Authors:** Pablo M De Salazar, Anne Marie Valadere, Christin H Goodman, Barbara W Johnson

**Affiliations:** 1 Caribbean Public Health Agency Caribbean Public Health Agency Port of Spain Trinidad and Tobago Caribbean Public Health Agency, Port of Spain, Trinidad and Tobago.; 2 Centers for Disease Control and Prevention Division of Vector-Borne Diseases Fort CollinsColorado United States Centers for Disease Control and Prevention, Division of Vector-Borne Diseases, Fort Collins, Colorado, United States of America.

**Keywords:** Chikungunya virus, reagent kits, diagnostic, immunoassay, immunoenzyme techniques, fluorescence polarization immunoassay, Caribbean Region, Americas, Virus chikungunya, juego de reactivos para diagnóstico, inmunoensayo, técnicas para inmunoenzimas, inmunoensayo de polarización fluorescente, Región del Caribe, Américas

## Abstract

The emergence of chikungunya virus in the Americas means the affected population is at risk of developing severe, chronic, rheumatologic disease, even months after acute infection. Accurate diagnostic methods for past infections are essential for differential diagnosis and consequence management. This study evaluated three commercially-available chikungunya Immunoglobulin G immunoassays by comparing them to an in-house Enzyme-Linked ImmunoSorbent Assay conducted by the Centers for Disease Control and Prevention (Atlanta, Georgia, United States). Results showed sensitivity and specificity values ranging from 92.8% – 100% and 81.8% – 90.9%, respectively, with a significant number of false-positives ranging from 12.5% – 22%. These findings demonstrate the importance of evaluating commercial kits, especially regarding emerging infectious diseases whose medium and long-term impact on the population is unclear.

## RESUMEN

Como consecuencia de la aparición del virus del chikungunya en las Américas, la población afectada corre el riesgo de padecer reumatismos crónicos graves, aun meses después de la infección aguda. Es fundamental contar con métodos precisos para diagnosticar los antecedentes de la infección a fin de elaborar un diagnóstico diferencial y abordar las manifestaciones de la fase crónica. Se han estudiado tres inmunoensayos comercializados de detección de inmunoglobulinas G para el diagnóstico del chikungunya, comparándolos con el enzimoinmunoanálisis de adsorción (ELISA) propio. Los resultados señalan valores de sensibilidad del 92,8% al 100% y de especificidad del 81,8% al 90,9%, así como un número significativo de falsos positivos, de entre el 12,5% y el 22%.

Local transmission of chikungunya virus (CHIKV) was first reported in the Americas in December of 2013 on the island of Saint Martin (1). The virus spread rapidly throughout the Caribbean and to the continental Americas. By the end of 2015, over 1.7 million cases had been reported by more than 45 countries in the Americas (2). Though severe and fatal cases of this mosquito-borne arbovirus (family *Togaviridae*, genus *Alphavirus*) were reported during the outbreak (3), typical acute infection ranges from mild to moderate symptomatology with complete recovery in 2 – 4 weeks (4). However, a certain proportion of patients later present with sub-chronic and chronic signs and symptoms, such as chronic inflammatory rheumatism with incapacitating and recurring episodes of polyarthritis and polyarthralgia (5, 6). In some cases, especially in the elderly, post-chikungunya chronic inflammatory rheumatism can persist for more than a year after the acute infection. Laboratory confirmation with accurate, differential diagnostic testing that excludes other rheumatologic diseases is essential to providing adequate management (7, 8).

Detection of the virus in patients’ serum by nucleic acid methods is limited to the first 5 –10 days, and Immunoglobulin M (IgM) is thought to be detectable in the bloodstream for no longer than 3 months after symptom onset (4). Thus, plaque reduction neutralization assays or detection of Immunoglobulin G (IgG) antibodies are currently the only methods used to confirm previous CHIKV acute infection in suspected chronic cases and in patients who did not seek medical attention during the disease’s acute phase. In-house validated CHIKV neutralization assays are time-consuming and difficult to implement, especially in limited-resource settings. In addition, few commercial CHIKV IgG detection assays are currently available. Thus, evaluation of commercial IgG detection kits is critical to increasing the capacity for accurate diagnosis of past CHIKV infection.

In this study, three commercially-available assay test kits—two enzyme immunoassays (EIA) and one immunefluorescence antibody technique (IFA)— that detect IgG antibodies against CHIKV were evaluated. Specifically, the assays chosen were the InBios IgG EIA (InBios International Inc., Seattle, Washington, United States) and the Euroimmun EIA and the Euroimmun IgG IFA (Euroimmun Medizinische Labordiagnostika AG, Luebeck, Germany).

## MATERIALS AND METHODS

### Clinical samples

The panel consisted of a total of 36 serum samples, 30 of which were from fever patients. Of these, 20 were suspected CHIKV cases whose samples had been sent to the laboratory of the Caribbean Public Health Agency (CARPHA) for confirmation during the 2014 CHIKV outbreak in the Caribbean. The outbreak specimens had been sampled 15 – 90 days after symptom onset. Another 10 samples predated the outbreak and were negative for dengue antibodies. The remaining six samples also predated the outbreak, but had been found positive for dengue antibodies and were included to assess cross-reactivity in specimens from patients with potentially similar clinical presentation. Aliquots of all specimens were stored at -80°C until further immunoassay testing commenced.

### In-house IgG assay

The aliquots of the 36 specimens were shipped to the Centers for Disease Control and Prevention (Atlanta, Georgia, United States; CDC), specifically to the Division of Vector-borne Diseases Arboviral Diseases Diagnostic and Reference Laboratory in Fort Collins, Colorado, for CHIKV IgG capture enzyme-link immunoassay (ELISA) testing, described elsewhere (9). CDC results were considered to be the reference standard.

### Commercial chikungunya IgG assays

The panel of 36 serum samples was tested using the aforementioned test kits—the InBios IgG EIA, the Euroimmun IgG EIA, and the Euroimmun IgG IFA. Testing was performed according to the manufacturers’ instructions. The cut-off dilution used for Euroimmun IgG IFA was 1/100.

### Statistical methods

For the purposes of this evaluation, test results were categorized as either CHIKV IgG positive and CHIKV IgG negative. Equivocal results were coded as negative for the analysis. Sensitivity was defined as the proportion of samples with a CDC reference standard result of CHIKV IgG positive that also had a CHIKV-positive test kit result. Specificity was defined as the percentage of reference standard CHIKV IgG negative results that also had a CHIKV IgG-negative kit result. The 95% confidence intervals (95%CI) were calculated with Wilson score interval continuity corrected. Accuracy was defined as the agreement of results between the evaluated kit and the reference standard assay.

## RESULTS

From the 36 samples sent to the CDC for testing, 38.8% (*n* = 14) were found to be positive, 52.7% (*n* = 19) were negative, and 8.3% (*n* = 3) had equivocal results (Table 1).

**TABLE 1.** Results of serologic diagnostic testing of 36 serum samples compareing three commercially-available chikungunya virus IgG immunoassays^a^ to the in-house results of the Centers for Disease Control and Prevention (Atlanta, Georgia, United States; CDC), 2015

Sample number	CDC in-house IgG	InBios EIA IgG	Euroimmun EIA IgG	Euroimmun IFA IgG
1	Positive	Positive	Positive	Positive
2	Positive	Positive	Positive	Positive
3	Positive	Positive	Positive	Positive
4	Positive	Positive	Positive	Positive
5	Positive	Positive	Positive	Positive
6	Positive	Positive	Positive	Positive
7	Positive	Positive	Positive	Positive
8	Positive	Equivocal^b^	Positive	Positive
9	Positive	Positive	Positive	Positive
10	Positive	Positive	Positive	Positive
11	Positive	Positive	Positive	Positive
12	Positive	Positive	Positive	Positive
13	Positive	Positive	Positive	Positive
14	Positive	Positive	Positive	Positive
15	Equivocal	Positive^b^	Positive^b^	Positive^b^
16	Equivocal	Positive^b^	Positive^b^	Positive^b^
17	Equivocal	Negative^b^	Positive^b^	Negative^b^
18	Negative	Negative	Equivocal^b^	Negative
19	Negative	Negative	Positive^b^	Negative
20	Negative	Negative	Equivocal^b^	Negative
21	Negative	Negative	Negative	Negative
22	Negative	Negative	Negative	Negative
23	Negative	Negative	Negative	Negative
24	Negative	Negative	Negative	Negative
25	Negative	Negative	Negative	Negative
26	Negative	Negative	Negative	Negative
27	Negative	Negative	Negative	Negative
28	Negative	Negative	Negative	Negative
29	Negative	Negative	Negative	Negative
30	Negative	Negative	Negative	Negative
31 D^c^	Negative	Negative	Negative	Negative
32 D	Negative	Negative	Negative	Negative
33 D	Negative	Negative	Negative	Negative
34 D	Negative	Negative	Negative	Negative
35 D	Negative	Negative	Negative	Negative
36 D	Negative	Negative	Negative	Negative

***Source:*** Prepared by the authors from the study data.

a InBios IgG enzyme immunoassays (EIA) manufactured by InBios International Incorporated (Seattle, Washington, United States), the Euroimmun EIA by Euroimmun Company, (Luebeck, Germany), and the IgG immune fluorescence antibody technique (IFA) also by Euroimmun.

b Discordant result compared to CDC reference standard.

c Previously tested positive for dengue antibodies.

As shown in Table 2, overall accuracy of the InBios IgG kit with CDC results was 91.7%, with 92.8% sensitivity (95%CI = 64.1% – 99.6%) and 90.9% specificity (95%CI = 69.3% – 98.4%); of these results, 13.3 % were false positive and 4.8% were false negative. The Euroimmune EIA showed overall accuracy of 88.8 %, with a sensitivity of 100% (95%CI = 73.2% – 100%) and specificity of 81.8 % (95%CI = 58.9% – 94%); of these results, 22.2% were false positives and none were false negative. The Euroimmune IFA showed an overall concordance of 94.4%, with a sensitivity of 100% (95%CI = 73.2% – 100%) and specificity of 90.9% (95%CI = 69.3% – 98.4%); of these results, 12.5% were false positive and none were false negative.

**TABLE 2.** Summary evaluation of the three commercially-available chikungunya virus IgG immunoassays,^a^ 2015

Test kit	Accuracy	Sensitivity (95%CI)	Specificity (95%CI)	FP^c^	FN^d^
InBios EIA	91.7%	92.8% (64.1% – 99.6%)	90.9% (69.3% – 98.4%)	13.3%	4.8%
Euroimmun EIA	88.8%	100% (73.2% – 100%)	81.8 % (58.9% – 94%)	22.2%	—
Euroimmun IFA	94.4%	100% (73.2% –100%)	90.9% (69.3% – 98.4%)	12.5%	—

***Source:*** Prepared by the authors from the study data.

a InBios IgG enzyme immunoassays (EIA) manufactured by InBios International Incorporated (Seattle, Washington, United States), the Euroimmun EIA by Euroimmun Company, (Luebeck, Germany), and the IgG immune fluorescence antibody technique (IFA) also by Euroimmun.

b 95% Confidence Interval.

c Percent of false positives compared to Centers for Disease Control and Prevention (Atlanta, Georgia, United States; CDC) reference standard.

d Percent of false negatives.

None of the commercial kits nor the in-house CDC assay showed cross-reactivity with the samples positive for dengue antibodies (Table 1).

## DISCUSSION

CHIKV spread extensively through-out Central and South America during 2014. As a result, a high proportion of the population in these areas is at risk of developing chronic inflammatory rheumatism, which can lead to persistent incapacitation (4, 5). Correct diagnosis and management requires affordable and reliable laboratory testing tools.

This comparison of three commercially-available kits for detection of IgG antibodies against CHIKV to the CDC in-house CHIKV IgG ELISA, showed acceptable sensitivity (92.8% – 100%) and specificity (81.8% – 90.9%). However, the significant number of false-positives (12.5% – 22%), particularly with the EIAs, indicates that further evaluations are needed to fully understand the limitations of the assays for clinical use.

To our knowledge, very few published studies have assessed commercially-available tools and verified protocols for diagnostics of CHIKV infection, especially IgG antibody detection. However, our results are consistent with previous evaluations (10). Although this study included only a small number of samples, its findings demonstrated the importance of evaluating commercial kits, especially when the medium and long-term impact of an emerging disease is unclear.

## Disclaimer.

Authors hold sole responsibility for the views expressed in the manuscript, which may not necessarily reflect the opinion or policy of CARPHA, CDC, the *RPSP/PAJPH* and/or PAHO.
